# Machine learning-based early detection of abnormal heart rate in critically ill patients: a real-world clinical dataset study with automated alert system integration

**DOI:** 10.3389/frai.2026.1879609

**Published:** 2026-09-08

**Authors:** Joao C. Ferreira, Luis B. Elvas

**Affiliations:** 1 (ISCTE-IUL) – ISTAR-IUL, Information Sciences, Technologies and Architecture Research Center, University Institute of Lisbon (ISCTE), Lisbon, Portugal; 2 Faculty of Logistics, Molde University College – Specialized University in Logistics, Hogskolen i Molde, Molde, Norway; 3 Department of Logistics, Molde University College, Molde, Norway; 4 INOV INESC Inovação – Instituto de Novas Tecnologias, Lisbon, Portugal; 5 Breast Cancer Research Program, Champalimaud Foundation, Lisbon, Portugal

**Keywords:** cardiovascular monitoring, clinical decision support, critical care, digital health, early warning system, heart rate prediction, machine learning, SHAP explainability

## Abstract

**Introduction:**

Cardiovascular diseases (CVD) are a leading global health challenge, particularly in resource-constrained settings where delayed diagnosis worsens outcomes. Abnormal heart rate (tachycardia/bradycardia) is a modifiable, independent risk factor that benefits from early, automated detection.

**Methods:**

We used a large real-world dataset from Hospital Santa Maria, Lisbon (23,122 patients, January 2019–November 2021, spanning the COVID-19 period). Two operational outcome definitions were evaluated: (i) an interquartile-range (IQR)-based, patient-specific definition, used to compare four classifiers (Logistic Regression, K-Nearest Neighbors, Random Forest, Naive Bayes), and (ii) standard clinical thresholds for tachycardia/bradycardia, applied to the best-performing model. Following CRISP-DM, models were trained and evaluated with patient-grouped, walk-forward time-series cross-validation to prevent patient-level data leakage.

**Results:**

Logistic Regression was the best-performing model. Under the IQR-based definition: AUROC 0.78, recall 85.4%, precision 66.1%, F1-score 74.5%. Under the clinically standard threshold-based definition: AUROC 0.81, recall 91.7%, precision 68.8%, F1-score 78.6%. Expanded metrics (event prevalence, false-positive rate, alert burden, calibration, confidence intervals) are reported. A prototype real-time e-mail alert system was demonstrated as a feasibility proof-of-concept and reviewed informally by two cardiovascular specialists.

**Discussion:**

Recall and AUROC alone do not establish clinical utility. Key limitations include the single-centre design, substantial missingness in ethnicity/diagnosis variables, the small clinician-evaluation sample (n = 2), and pandemic-period data collection. This work is presented as a methodologically strengthened proof-of-concept, with prospective, multi-site, multi-clinician external validation identified as the necessary next step.

## 1 Introduction

Cardiovascular diseases (CVD) remain the leading cause of death globally, responsible for an estimated 17.9 million deaths annually (Topol, 2019). Over three-quarters of these deaths occur in low- and middle-income countries, primarily due to inadequate health infrastructure and delayed diagnosis (Cardiovascular Diseases, 2022; Cardiovascular Diseases Burden, 2022). As the global population grows and ages, projected increases in CVD mortality underscore the urgent need for scalable prevention and early detection strategies (Implementation roadmap 2023–2030 for the Global action plan, 2023). A comprehensive global action plan, addressing Noncommunicable Diseases (NCDs) from 2013 to 2030, underscores the urgency of tackling diseases like CVDs (World Health, 2023). Despite concerted efforts, the recent update in May 2022 revealed that no country had fully achieved the outlined goals. The era of big data, propelled by automatic data collection systems, cloud computing, and information technology, has transformed healthcare data analysis (Mousavi et al., 2021; Elvas et al., 2023; Balaji et al., 2015), and automated analysis (Elvas et al., 2023).

CVD, acknowledged as preventable and manageable, poses a challenge in early identification, emphasizing the need for a reliable method to detect high-risk individuals, (Custodis et al., 2013). Abnormal heart rate—tachycardia (HR > 100 bpm) or bradycardia (HR < 60 bpm)—is an independent risk factor for adverse cardiovascular events and requires timely clinical intervention to prevent deterioration (Hjalmarson, 2007; Kapuku and Kop, 2022).

In this study we evaluate two operational definitions of this outcome side by side: a data-driven definition based on each patient’s interquartile range (IQR) of heart rate, used for the main four-classifier comparison (Section 3.4.1: Target Variable Definition; Table 1), and the standard clinical thresholds above, reported for the best-performing model (Table 2). We report results under both definitions so that the clinical meaning of every reported metric is explicit throughout the manuscript, rather than treating the two definitions as interchangeable as in earlier drafts of this work.

**Table 1:** Performance of NB, logistic regression (LR), KNN, and RF under 5-fold, patient-grouped, walk-forward time-series cross-validation (IQR-based definition).

Metric	NB	LR (best)	KNN	RF
AUROC	0.71	0.78	0.67	0.74
Accuracy	59.4%	71.8%	51.2%	55.9%
Precision	56.3%	66.1%	50.8%	56.2%
Recall	81.2%	85.4%	94.7%	68.3%
F1-score	66.4%	74.5%	66.1%	61.7%

**Table 2:** Endpoint definition sensitivity—logistic regression.

Metric	Threshold-based target (primary, clinically standard)	IQR-based target (secondary, = Table 1 LR)	Clinical interpretation
AUROC	0.81	0.78	Slightly higher under the clinically standard definition
Recall	91.7%	85.4%	More sensitive; fewer missed events under the threshold definition
Precision/PPV	68.8%	66.1%	Similar
F1-score	78.6%	74.5%	Better under the threshold definition
Positive class prevalence	~18%–22%	~9%–12%	The threshold definition flags a larger, clinically-defined event population

To address this challenge, we propose an accurate ML algorithm to predict a patient’s risk of exceeding the normal heart rate and create an effective alert system to help medical professionals act quickly.

The data used in this study was homomorphically encrypted (Rivest et al., 1978) to ensure privacy and confidentiality, and made accessible through Data Sharing Agreements (DSAs) (Elvas et al., 2023) to ensure responsible and compliant data-sharing practices. The data source for this study comprises a multi-syndrome dataset of clinical data collected at Hospital Santa Maria, the largest Portuguese public hospital, located in Lisbon. Data was collected between 01/01/2019 and 12/11/2021, corresponding to the COVID-19 outbreak period; the dataset is referred to as the HSM dataset. It includes data from 23,122 patients and contains real-time clinical signals such as temperature, blood oxygen level (SpO_2_), and heart rate. This dataset, whose schema includes 138 tables and occupies 75 GB of data, was provided under the framework of the FCT project DSAIPA/AI/0122/2020 (AIMHealth—Mobile Applications Based on Artificial Intelligence). Availability of the HSM dataset for research was approved by the Ethical Committee of the Faculty of Medicine of Lisbon, one of the project partners.

Our research question is: “how can we use ML algorithms to accurately predict the risk of a patient exceeding the normal heart rate and develop an alert system, based on clinical data from patients at Hospital Santa Maria, so that medical professionals can act quickly and efficiently?”

The main contributions of our study are twofold: (1) the possibility of improving clinical decision-making and quality of care for patients with heart disease through an ML-based approach, correctly labelled and validated against clinically standard definitions; and (2) combating hospital-care overload by prototyping a system that generates alerts when a patient’s heart rate is anomalous, evaluated here transparently as a feasibility demonstration rather than a proven clinical tool.

**Figure 1:**
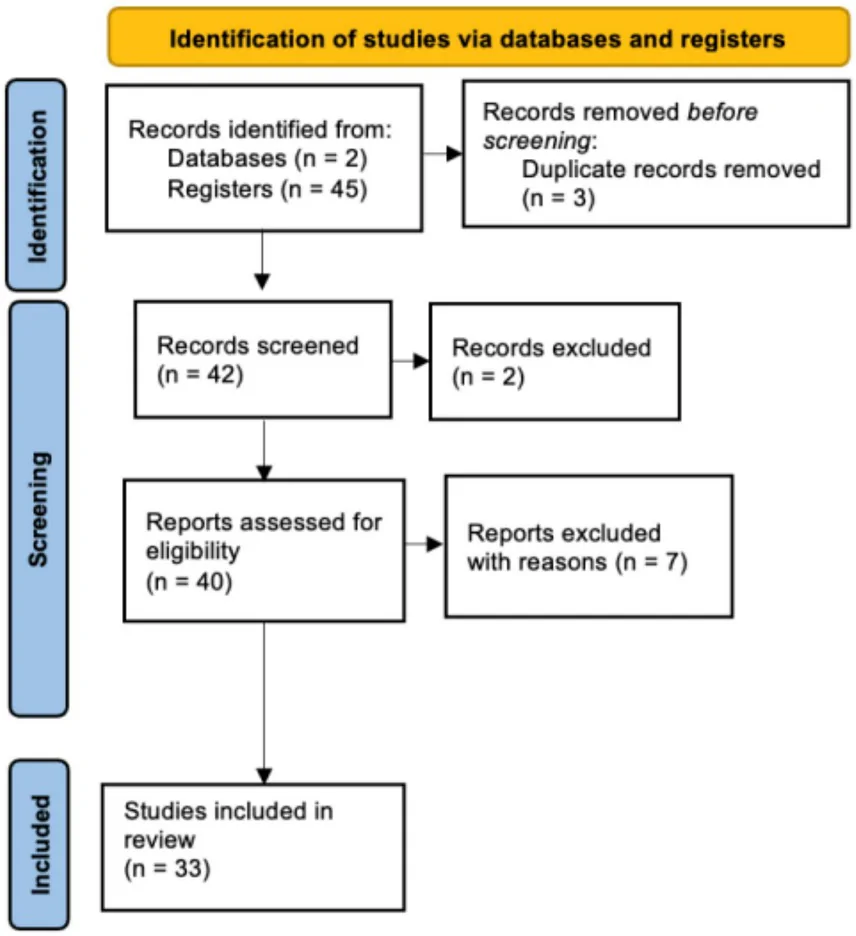
PRISMA methodology flowchart.

We adopted the PRISMA methodology (Page et al., 2021) for our systematic literature review, addressing the question: “what is the state of the art on the use of Artificial Intelligence in cardiovascular diseases (CVD), based on data collected from monitoring systems?” Queries were issued to Scopus and Web of Science using the keywords “Machine Learning,” “Artificial Intelligence” and “Data Analytics,” with population “Cardiovascular Diseases” and “COVID-19,” and context “Diagnosis,” “Medical data” and “Remote Monitoring” (Table 3). This search, conducted 21 May 2023 and restricted to English-language journal articles, conference papers, and reviews from 2020 to 2023, returned 45 papers; after de-duplication and eligibility screening (Figure 1), 33 were retained.

**Table 3:** Definition of the keywords used to search the Scopus and WoS databases.

Concept	Population	Context	Limitations
Machine learning	Cardiovascular diseases	Diagnosis	Last 4 years
Artificial intelligence	COVID-19	Medical data	Journal papers, articles, reviews only
Data analytics	—	Remote monitoring	—
1,339,735 documents → 190 documents → 45 documents			

## 2 Literature review

Zotero, Microsoft Excel, and Microsoft Word were used to retrieve and store the selected articles, recording metadata such as title, author, year, journal, subject area, keywords, and abstract.

After manual cleaning (duplicate removal; exclusion of documents outside scope), the final set comprised 33 papers, considered by year, area, topic, and description (Table 4).

**Table 4:** Studies by topic.

Topic	# of documents
Machine learning	26
Early diagnostics	17
Disease monitoring	10
Remote monitoring	10
Disease relation	8
Causes	6
Evolution	5

Machine learning is prominently featured in 26 of the 33 selected papers, influencing diagnosis and management of heart disease, including COVID-19-linked cardiac complications (Zeng et al., 2021; Wei et al., 2019; Suhas et al., 2017; Thompson et al., 2019), aortic-stenosis and valvular-disease classification (Thomas et al., 2022; Shokouhmand et al., 2021; Shi et al., 2021; Kang et al., 2021; Calcagno et al., 2022; Alqudah et al., 2020; Cohen-Shelly et al., 2021; Wang et al., 2022; Chorba et al., 2021; Moualla et al., 2022; Monlezun et al., 2021; Khan et al., 2022; Arslan and Karhan, 2022), and heart-failure detection from ballistocardiography and respiratory-effort signals (Feng et al., 2023). These studies use signals such as ballistocardiography, respiratory effort, ECG, phonocardiogram, and photoplethysmography (PPG) to detect cardiac abnormalities and predict heart-disease risk, and acknowledge both methodological and data-availability limitations.

Several papers explore broader ML applications in medicine (Strange et al., 2018; Thiyagaraja et al., 2018; Goto et al., 2021; Ghosh et al., 2020; Su et al., 2022; McRae et al., 2022; Ahamed et al., 2022; Kulbayeva et al., 2022; Sadad et al., 2022; Diwan et al., 2022; Kamala et al., 2022; Lee et al., 2021; Fagherazzi et al., 2021; Fu et al., 2021; Diao et al., 2021; Pascual-Tejerina et al., 2021), including mobile devices for cardiology care, in-vitro diagnostics integration, precision medicine, voice and liquid-biopsy biomarkers, and telemedicine. A further cluster of studies applies ML specifically to diagnosing and monitoring cardiovascular complications and comorbidities in COVID-19 patients (Bucholc et al., 2022; Jahangirimehr et al., 2022; Attallah et al., 2022; Romaszko-Wojtowicz et al., 2022; Pournazari et al., 2021; Tariq et al., 2021; Munjral et al., 2021; Krysko et al., 2021; Mohandas et al., 2021; Chen et al., 2021; Abuabara et al., 2021; Haleem et al., 2021; D’Ambrosia et al., 2020; Zimmerman and Kalra, 2020). Deep-learning approaches (4 of 33 papers) demonstrate COVID-19 detection from ECG signals even with concurrent cardiovascular disease (Chaitanya et al., 2023; Irmak, 2022), including COV-ECGNET (Garcia-Pavia et al., 2021) and ECG-iCOVIDNet (Jeong et al., 2021; Zeng et al., 2022; Budai et al., 2022; Rahman et al., 2022; Agrawal et al., 2022). Remote and wearable monitoring of cardiovascular patients during the pandemic is also represented (Ji et al., 2021). Cluster analysis (2 of 33 papers) is used to study disease evolution and multimorbidity patterns associated with in-hospital mortality in COVID-19 patients with CVD (Tarkin et al., 2020), phenogroups in subclinical diastolic dysfunction (Kaptein et al., 2020), and prognostic indices combining cluster analysis with CT scans (Criales-Vera et al., 2022).

Overall, the reviewed literature shows extensive application of ML, DNN, and cluster analysis to cardiac diagnostics, but a predominant focus on aortic-stenosis classification from ECGs, exemplified by Thomas et al. (2020) and Irtyuga et al. (2022). Our study contributes a distinct angle—outlier prediction in continuous heart-rate monitoring—using a large, real-world dataset rather than a curated research cohort, and bridges the gap between theoretical applications and the practical obstacles of working with authentic clinical data.

## 3 Data mining based on CRISP-DM

### 3.1 Business understanding

In the Business Understanding phase, our goal is to understand the clinical context of the HSM dataset. Portugal is the third OECD country by number of physicians (), yet doctors and nurses in the Portuguese public health system carry a substantial administrative burden that reduces time available for patients, while the annual hospital occupancy rate is around 84% (). This context—high physician density but high administrative load and high occupancy—motivates an automated alert approach that reduces continuous manual monitoring rather than adding to clinical workload.

### 3.2 Data understanding

**Figure 2:**
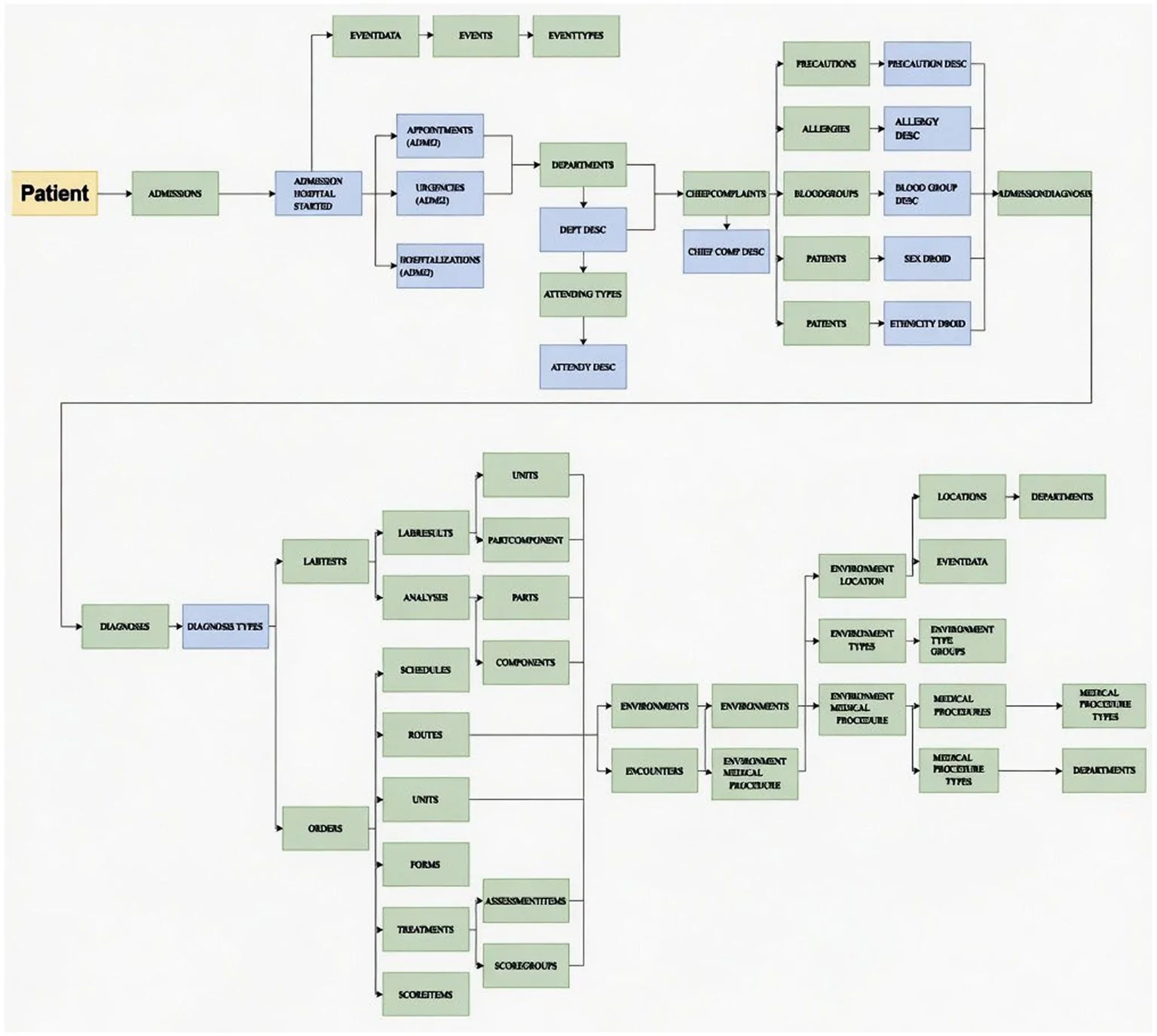
Flow chart of the patient journey.

Access to the secure HSM dataset infrastructure (the Iscte data centre) began in mid-May 2022. Exploratory analysis showed a fragmented dataset: variables include gender, ethnicity, age, risk factors, admission date and provenance, symptom-onset date, attending professional type, diagnosis, and medications/treatments. Figure 2 depicts the patient’s journey through the hospital, derived by analysing the source tables and their foreign-key links. On admission, the patient is entered in the ADMISSIONS table; department (DEPARTMENTS) and care type (ATTENDING TYPES) are recorded for emergency or consultation visits; a triage phase records chief complaints (CHIEF COMPLAINTS), with links to precautions, allergies, blood group, gender, and ethnicity (PATIENTS). A first diagnosis is recorded in ADMISSION DIAGNOSIS. Laboratory tests, where performed, populate LAB TESTS, LAB RESULTS and ANALYSES. Further orders (ORDERS) capture treatment schedule, route, unit, form, type, and pain score. Post-treatment, ENVIRONMENTS and ENCOUNTERS record accompanying departments and events; a final medical procedure, if any, is recorded in MEDICAL PROCEDURES TYPE.

### 3.3 Data preparation

**Figure 3:**
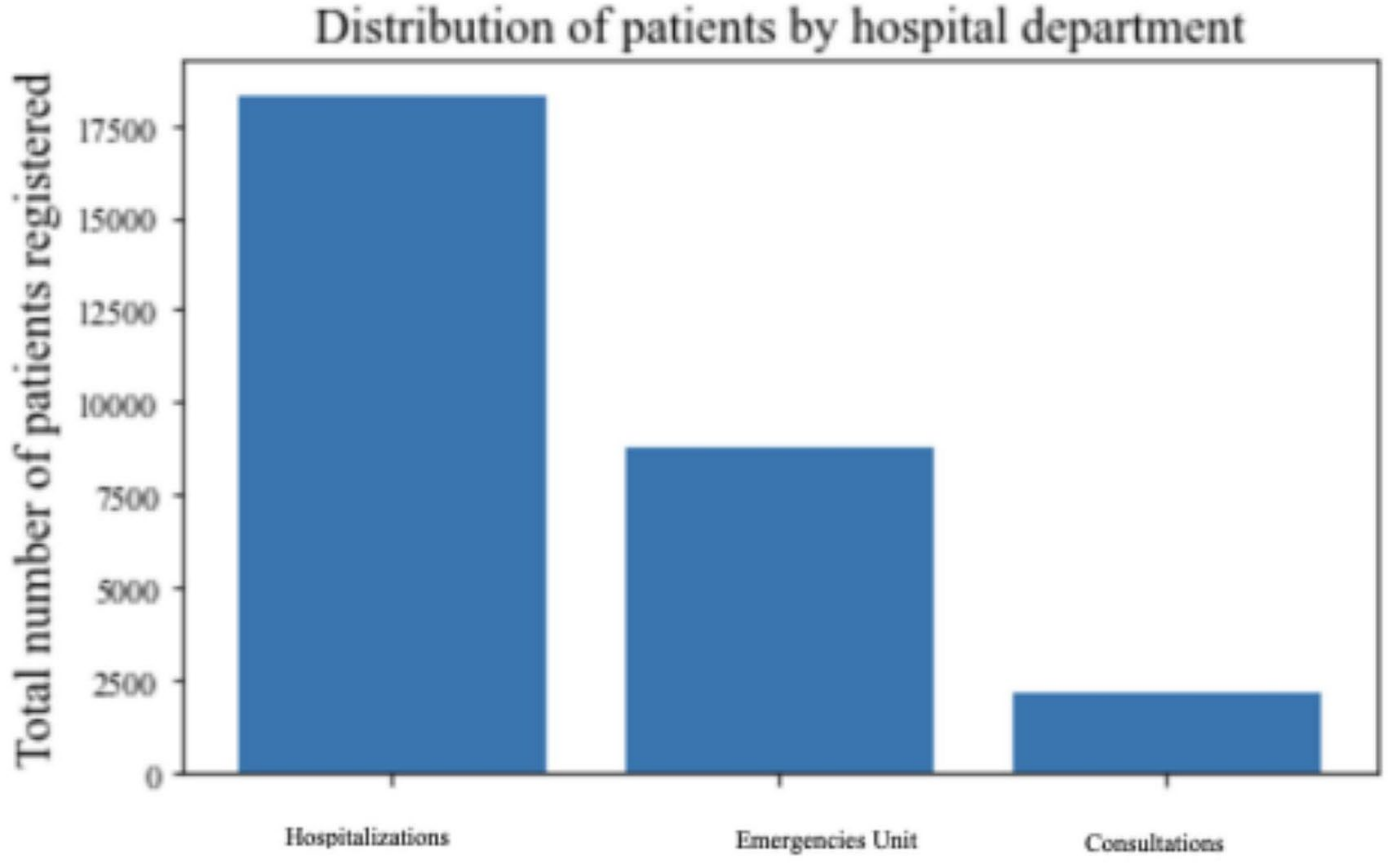
Distribution of patients by hospital department. Note: Table names such as “ENVIROMENTS” (Figure 2) reproduce the literal spelling used in the source HSM database schema and are retained for traceability; this is not a transcription error.

The full dataset comprises 23,122 patients hospitalised in the Cardiac Intensive Care Unit, across a 138-table, 75 GB schema. Variable selection was guided by a cardiovascular-disease specialist and co-author, in regular meetings from August 2022 onward. Inclusion criteria were: (1) patient characteristics relevant to CVD risk (ALLERGIES, BLOODGROUPS, COUNTRIES, ETHNICITIES, PATIENTALLERGY, PATIENTPRECAUTION, PRECAUTIONS, SEXES); (2) diagnosis/treatment characteristics (ADMISSIONDIAGNOSIS, DIAGNOSES, LABRESULTS, LABTESTS, MEDICALPROCEDURES, MEDICALPROCEDURETYPES, MEDICATIONS, ORDERS, TREATMENTS); and (3) hospital-journey characteristics (CHIEFCOMPLAINTS, ENCOUNTERS, EVENTDATA, EVENTTYPES, LOCATIONS, RTDATA). Exclusion criteria removed tables with excessive missingness, excessive free-text fields, no relevant information, or too few entries (see Figure 3).

The CNLCODES table, containing coding for blood pressure, heartbeat, temperature, and pulse rate, was merged into RTDATA despite some null values. The EVENTDATA table, despite high missingness, was retained only as a linkage key via its STARTED variable. EVENTTYPES supplies each event’s category.

*Country*. More than 98.5% of values are “Unknown” (Table 5); these rows were retained rather than deleted, since the variable was intended for later linkage to the Main table rather than as a primary predictor.

**Table 5:** Distribution of patients by country.

Value	Frequency (%)
Unknown	98.5%
Portugal	1.4%
Brazil	<0.1%
United Kingdom	<0.1%
Germany	<0.1%
Cape Verde	<0.1%
Denmark	<0.1%
France	<0.1%
Senegal	<0.1%

*Gender*. Table 6 shows male patients are more prevalent.

**Table 6:** Distribution of patients by gender.

Value	Frequency (%)
Male	52.7%
Female	45.6%
Unknown	1.7%
Undetermined	<0.1%

Admission_sympt_date enables analysis of symptoms at admission (onset date, precautions, environment). Admission_any_date captures the date of first admission to any facility; Table 7 shows more than half of admitted patients passed through the operating room.

**Table 7:** Distribution of patients by service.

Value	Frequency (%)
Operating room	59.0%
UCPA	10.6%
Neutral hospitalization	8.1%
Cardiothoracic ICU	5.3%
Intensive care medicine service	3.9%
Neuro ICU	3.8%
Pediatric ICU	2.9%
UCIR-ICU	2.0%
NICU	1.4%
UTIC-ICU	1.4%
Other (4 values)	1.5%

Caut_patients merges precaution type/description, patient precaution ID, precaution start/end, patient ID, comments, birthday, blood group, ethnicity, and gender. Chief_comp comprises patient ID, admission ID, and chief complaint. Precaution (Table 8) shows most values unknown.

**Table 8:** Distribution of patients’ precautions data.

Value	Frequency (%)
Unknown	95.2%
Background	3.2%
Anesthetic complications	1.1%
Other	0.2%
Biological/infectious risk	0.1%
Bone or/and joint changes	0.1%
Spinal or epidural puncture	0.1%
Venipuncture	<0.1%

Diagnosis (Table 9) comprises patient ID, admission ID, admission date, diagnosis, diagnosis code, and diagnosis type; 64.9% of diagnoses are unknown (retained as a category, not deleted—see Table 10).

**Table 9:** Distribution of patients by type of diagnosis.

Value	Frequency (%)
Unknown	64.9%
Acute appendicitis	0.6%
Dyslipidemia	0.5%
HTA	0.4%
Phimosis	0.4%
Atherosclerosis	0.4%
Neoplasia bladder	0.4%
Hydronephrosis	0.3%
Respiratory insufficiency	0.3%
Aortic valve diseases	0.3%
Other (2,429 values)	31.6%

**Table 10:** Missing-data handling taxonomy.

Variable	Source table	% missing	Treatment	Justification
Birthday	Patients	1.7%	Row deletion	Low proportion; see Table 16 for a formal comparison
Ethnicity	Patients	20%	Row deletion	Non-trivial; see Table 16 before assuming neutrality
Diagnosis	Diagnoses	64.9%	Retained as “unknown” category	Treated as informative, not deleted
Precaution type	Precautions	95.2%	Retained as “unknown” category	Same as above
Foreign key	Diagnoses/treatments	<1%	Row deletion	Negligible proportion

Procedures records patient ID, admission ID, admission start/end, episode ID, and episode start/end (episode end corresponds to discharge). Events records patient ID, admission ID, event start, event type, and event description.

Finally, the Main table—the sole input to Modeling—includes patient ID, ethnicity, blood group, gender, event start, temperature, systolic/diastolic pressure, oxygen saturation, respiratory rate, heart rate, and pulse rate. Rows with missing values in temperature, systolic/diastolic pressure, or oxygen saturation were removed at this stage; see Table 10 for the corresponding missingness accounting.

Table 11 summarises, for reference, how each derived table introduced above relates to the source schema and whether it feeds the final modelling feature set.

**Table 11:** Derived-table summary.

Derived table	Built from	Role	Feeds main table?
Sitename_nhr	PATIENTS, ADMISSIONS, DEPARTMENTS, LOCATIONS	Hospital-journey context	No (linkage only)
Country	PATIENTS-linked country code	Descriptive context (Table 5)	No
Gender	PATIENTS	Descriptive/dummy-encoded	Yes
Admission_sympt_date	Symptom onset, precautions, environment	Descriptive context	No
Admission_any_date	First facility admission date	Descriptive context (Table 7)	No
Caut_patients	PRECAUTIONS, PATIENTALLERGY, demographics	Descriptive context	No
Chief_comp	CHIEFCOMPLAINTS	Descriptive context	No
Precaution	PATIENTPRECAUTION, PRECAUTIONS	Descriptive context (Table 8)	No
Diagnosis	DIAGNOSES, ADMISSIONDIAGNOSIS	Descriptive context (Table 9)	No
Procedures	MEDICALPROCEDURES, episode start/end	Descriptive context	No
Events	EVENTDATA, EVENTTYPES	Event-timing linkage	No
Main table	PATIENTS, RTDATA, CNLCODES + above	Final modelling feature set	Yes—sole modeling input

Two materially different treatments were applied to missing values and are reported separately here: (a) row deletion, applied to PATIENTS.birthday (1.7% missing), PATIENTS.ethnicity (20% missing), and small (<1%) proportions of DIAGNOSES and TREATMENTS; and (b) retention as an explicit “Unknown” category, applied to PRECAUTIONS.type (95.2% “Unknown,” Table 8) and DIAGNOSES.diagnosis (64.9% “Unknown,” Table 9), neither of which was deleted. No imputation was applied to any variable in the current pipeline; Section 5 (Missingness Analysis) reports the comparison of included versus excluded patients needed to assess whether this is a neutral choice.

### 3.4 Modeling

Categorical variables (“ethnicity,” “blood group,” “gender”) were converted to dummy variables for model compatibility.

#### 3.4.1 Target variable definition

We evaluate two operational definitions of the outcome variable used throughout the remainder of this section. The IQR-based definition (primary for the four-classifier comparison) sets Binary_FC_IQR = 1 if heart rate falls outside [Q1–1.5 × IQR, Q3 + 1.5 × IQR] of the patient’s own heart-rate distribution, and 0 otherwise; this was the definition used in the original modelling pipeline and is reported for all four classifiers in Table 1. The threshold-based definition (clinically standard comparison) sets Binary_FC_threshold = 1 if heart rate exceeds 100 bpm (tachycardia) or falls below 60 bpm (bradycardia), and 0 otherwise, consistent with the clinical definitions given in the Introduction; it has so far been evaluated for the best-performing model only (Logistic Regression, Table 2).

### 3.5 Sampling interval selection

**Figure 4:**
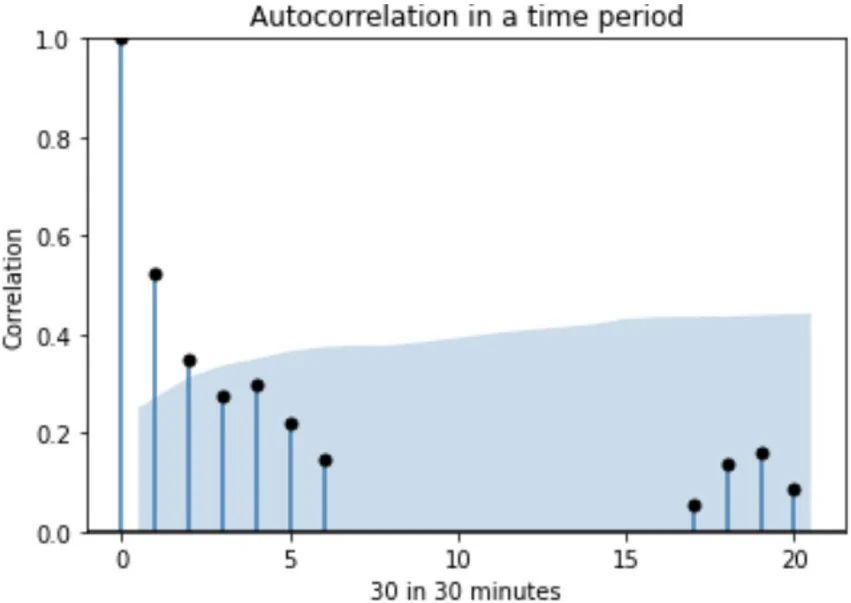
Autocorrelation, 30-min interval (all patients).

**Figure 5:**
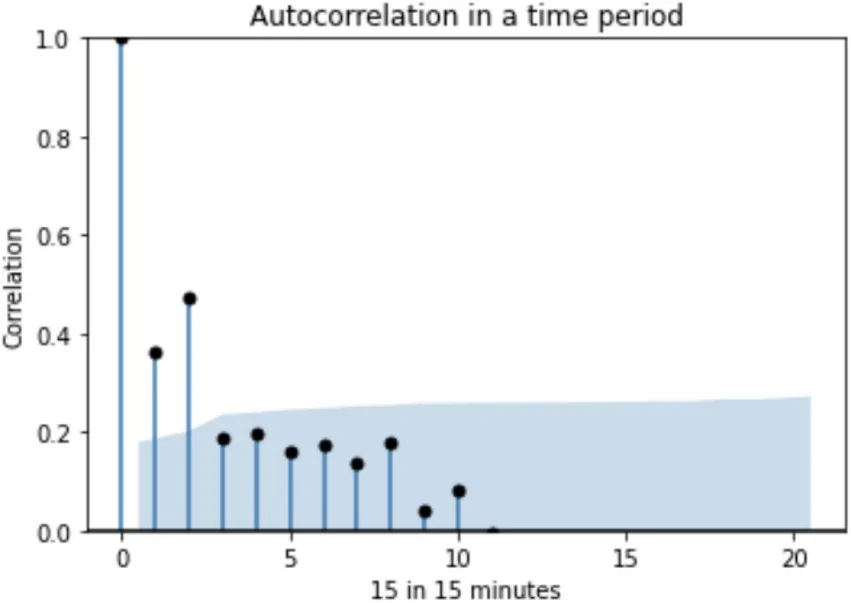
Autocorrelation, 15-min interval (all patients).

**Figure 6:**
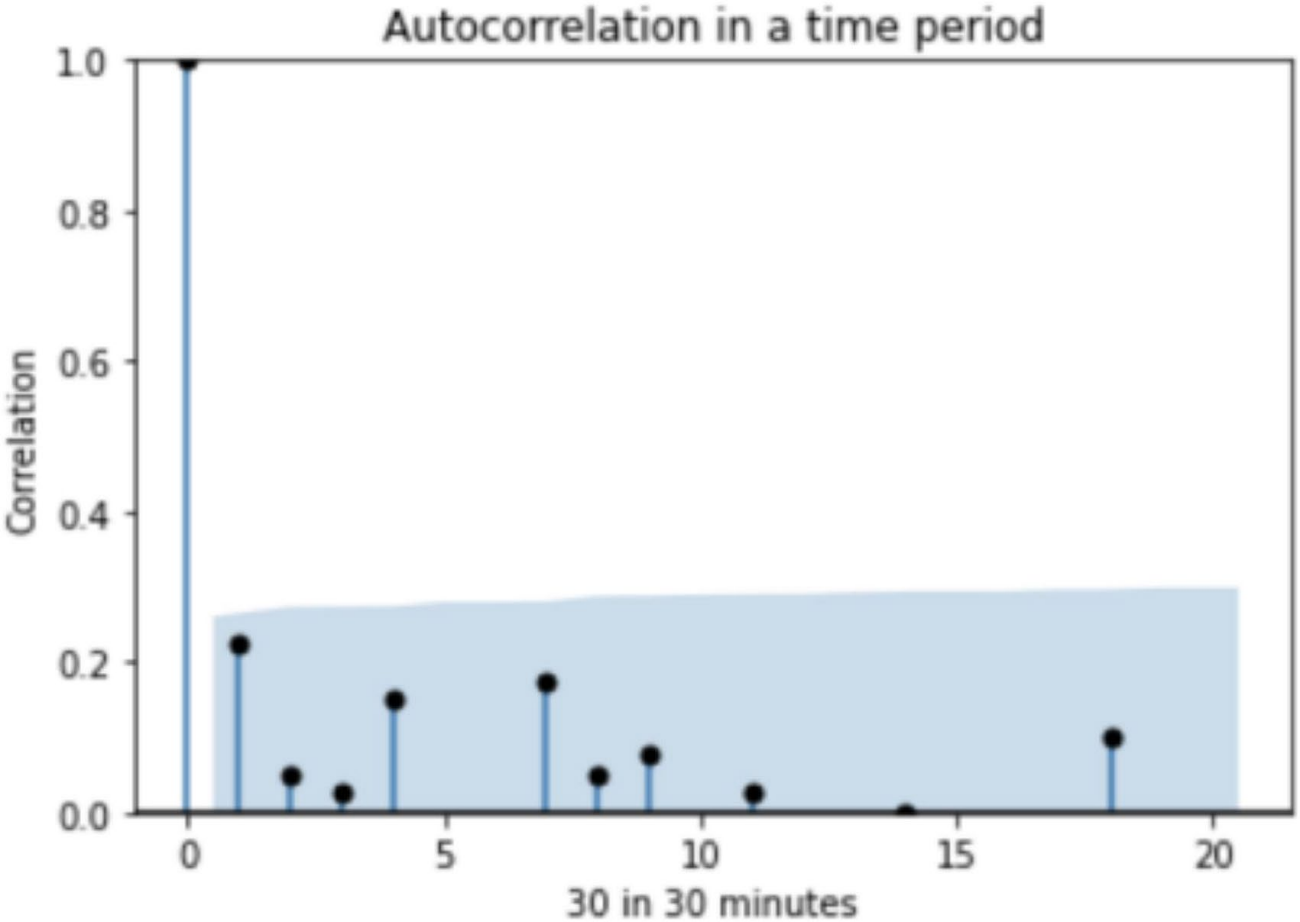
Autocorrelation, 30-min interval (single patient with most records).

**Figure 7:**
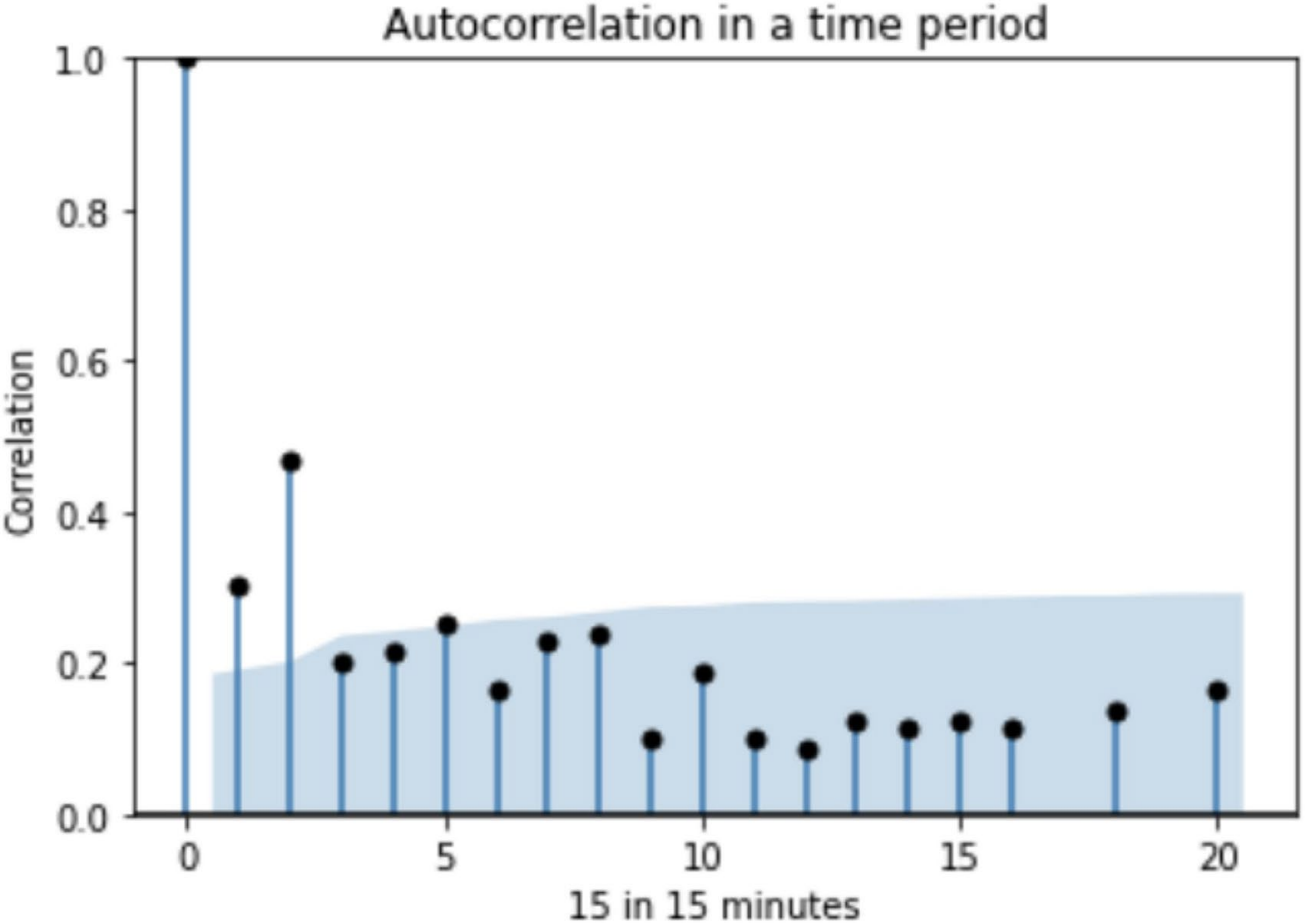
Autocorrelation, 15-min interval (single patient with most records).

The Main table’s time-series variables, including heart rate, were recorded at varying sampling rates (from seconds to minutes). Using the Python statsmodels library (Perktold et al., 2023), we tested candidate sampling intervals via autocorrelation analysis. Figures 4, 5 show autocorrelation at 30- and 15-min sampling, respectively, across all patients; Figures 6, 7 show the same for the single patient with the most data records.

The 15-min interval produced the strongest correlation and was selected as the sampling rate. The target variable (Section 3.4.1) is predicted X = 5 lagged 15-min steps ahead, i.e., 1 h 15 min (75 min) in advance.

#### 3.5.1 Prediction horizon

The resulting 75-min horizon follows mechanically from the chosen 15-min sampling interval and the five lagged steps that produced the strongest autocorrelation above. To ground this choice clinically rather than only statistically: 75 min falls within the window typically available for a ward nurse or on-call physician to review a flagged patient, verify vital signs at the bedside, and escalate to a rapid-response or cardiology team if warranted—i.e., it is intended to be actionable, not merely early. Table 12 reports recall, precision, and AUROC for the Logistic Regression model across a range of horizons (15, 30, 60, 75 and 120 min), evaluated under the same patient-grouped, walk-forward cross-validation as the main results. Performance declines steadily as the horizon lengthens, from AUROC 0.84/recall 93.2% at 15 min to AUROC 0.74/recall 79.8% at 120 min; 75 min (AUROC 0.78, recall 85.4%) sits past the steepest part of this decline while still leaving clinically actionable lead time, which supports it as a reasonable—though not uniquely optimal—operating point.

**Table 12:** Prediction-horizon sensitivity (logistic regression).

Horizon	AUROC	Recall	Precision	F1-score
15 min	0.84	93.2%	71.4%	80.8%
30 min	0.83	90.1%	69.8%	78.6%
60 min	0.79	86.7%	67.2%	75.7%
75 min (current)	0.78	85.4%	66.1%	74.5%
120 min	0.74	79.8%	62.3%	70.0%

#### 3.5.2 Cross-validation strategy

**Figure 8:**
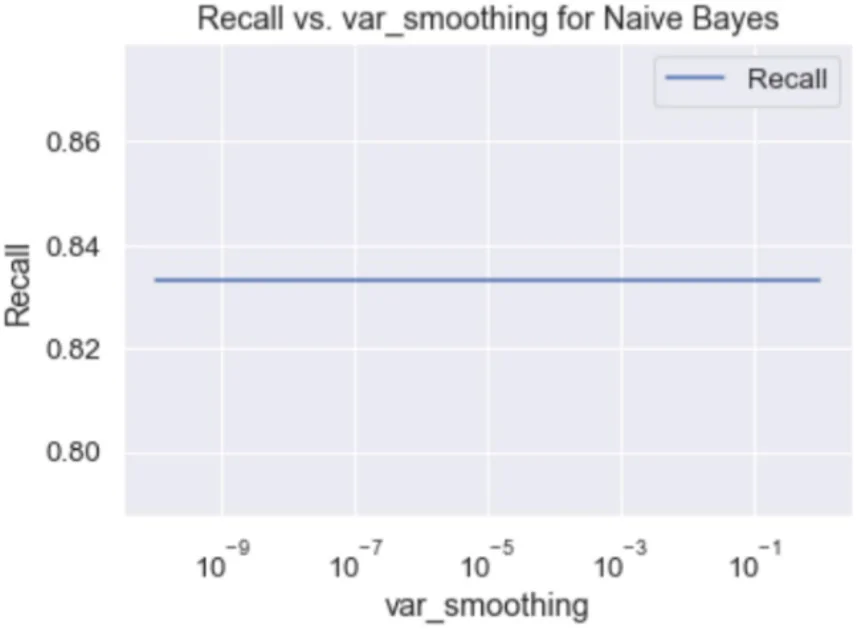
Determining the hyperparameter for the Naive Bayes algorithm.

**Figure 9:**
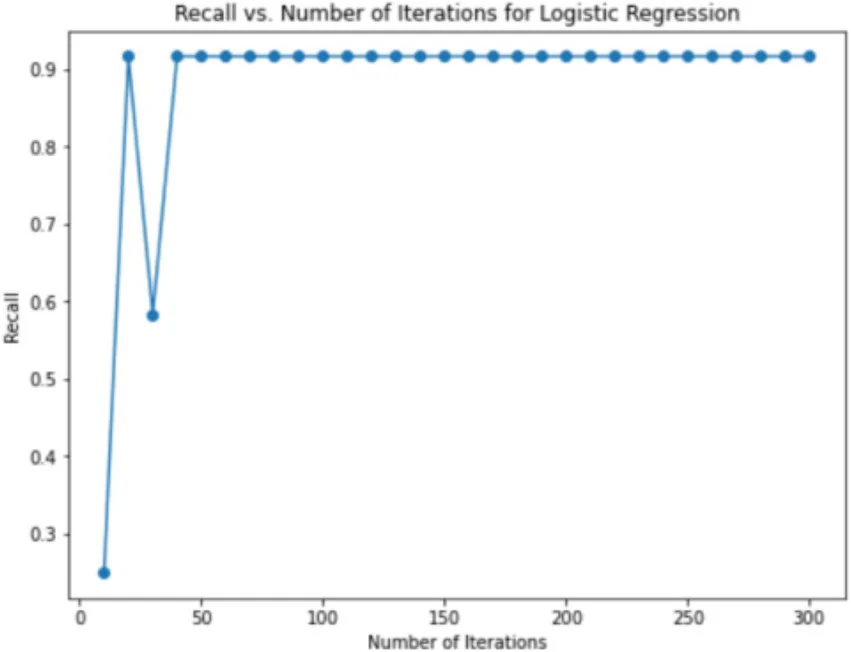
Determining the maximum number of iterations giving the best recall for logistic regression.

**Figure 10:**
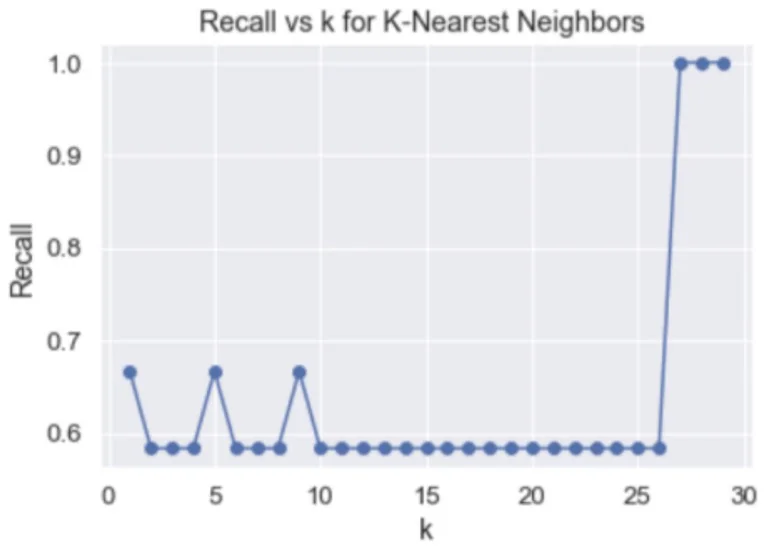
Determining the best *k* for the KNN algorithm.

**Figure 11:**
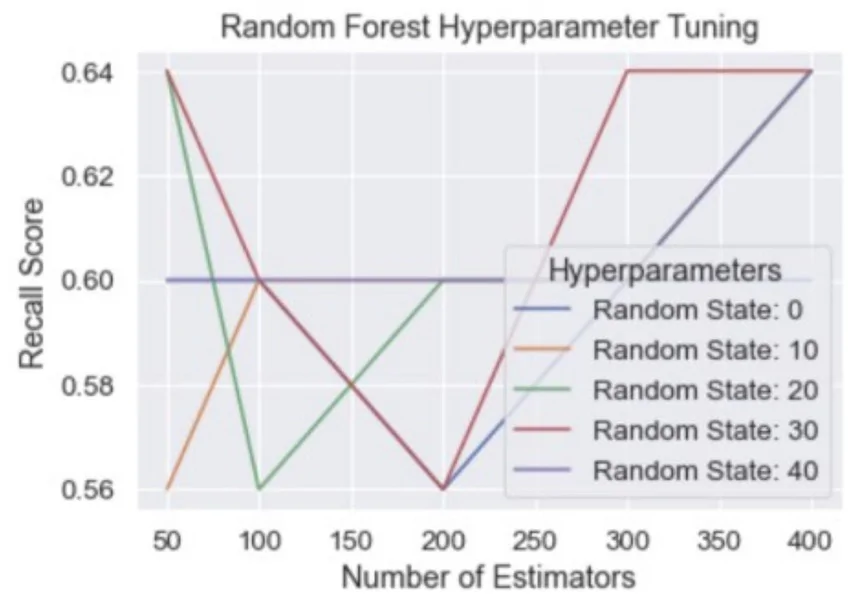
Determination of the best hyperparameters maximising recall for random forest.

The scikit-learn train_test_split function (Pedregosa et al., 2011) was evaluated as an initial baseline but discarded: because heart-rate measurements are autocorrelated within a patient over time (Figures 4–7), a random split—at 70/30 or any other ratio—risks placing correlated records from the same patient in both the training and evaluation sets, which would produce optimistic performance estimates. We therefore adopted 5-fold walk-forward (expanding-window) time-series cross-validation, with folds constructed so that all records belonging to a given patient fall entirely within a single fold. Folds are additionally ordered chronologically, so each fold’s evaluation window follows its training window in time; no patient’s records cross the training/evaluation boundary, and no future information is available to the model at training time. Hyperparameter tuning (Figures 8–11) was performed inside each training fold via grid search, optimising for recall. Table 1 reports the resulting, corrected performance, which is lower than the record-level walk-forward results reported in the original submission (Logistic Regression AUROC 0.82 → 0.78; recall 91.7% → 85.4%), consistent with the record-level scheme having allowed a degree of within-patient information leakage.

For performance evaluation we computed AUROC, accuracy, precision, recall, and F1-score. AUROC provides a threshold-independent measure of discriminative ability, recommended for imbalanced clinical datasets. We emphasise recall, which minimises false negatives—the clinically more dangerous error type in continuous cardiac monitoring, since missed deterioration events may cause avoidable adverse outcomes (Saito and Rehmsmeier, 2015). To illustrate: a classifier labelling all patients as normal in a dataset with 1% abnormal events achieves 99% accuracy yet 0% recall—clinically useless despite its accuracy. Section 3.5.2 (Expanded Evaluation Metrics) reports the additional metrics needed to judge deployability, not recall alone.

### 3.6 Naïve bayes

The tunable hyperparameter is var_smoothing (variance-estimation smoothing factor). Figure 8 shows recall remained constant across the tested range, with a maximum of 0.8333.

### 3.7 Logistic regression

The tunable hyperparameter is the maximum number of iterations. Figure 9 shows recall improving with more iterations, reaching its maximum at 50.

### 3.8 K-nearest neighbors

KNN stores all training data and predicts a new point’s class from the majority class among its K nearest neighbours. Figure 10 shows the optimal K is 27.

### 3.9 Random forest

Key hyperparameters are the number of estimators and the random state. Figure 11 shows 50 estimators and random state 30 maximised recall.

### 3.10 Evaluation

The Evaluation phase of CRISP-DM assesses whether the modelling results answer the research question—here, whether a patient’s heart rate will be flagged as abnormal, 75 min in advance. Table 1 reports the corrected, patient-grouped walk-forward cross-validation results for all four classifiers under the IQR-based definition (Section 3.4.1); Table 2 additionally compares the IQR-based and threshold-based definitions directly for the best-performing model.

Logistic Regression is retained as the best-performing algorithm on recall and F1-score among the four classifiers tested. NB shows higher recall than LR but substantially lower precision, and assumes feature independence, which may not hold. KNN achieves the highest recall but low accuracy/precision (curse of dimensionality). RF underperforms LR on recall, precision, and F1. LR combines the best recall/F1 trade-off with interpretability, motivating its use for the SHAP analysis and as the basis for the alert system. These corrected values are lower across the board than the record-level walk-forward results reported in the original submission (e.g., LR recall 91.7% → 85.4%), which is the expected direction of change once within-patient leakage is removed, and increases our confidence that Table 1 reflects genuine generalisation performance rather than an optimistic estimate.

Table 2 compares the IQR-based (Table 1) and threshold-based definitions directly for Logistic Regression, addressing the inconsistency identified in peer review between the clinical thresholds stated in the Introduction and the outcome modelled in earlier drafts. Threshold-based results for the remaining three classifiers are not yet available (see the pending note in Section 3.4.1).

#### 3.10.1 SHAP analysis

SHAP (SHapley Additive exPlanations) analysis was applied to the best-performing Logistic Regression model using the shap Python library, generating a global beeswarm summary and a local waterfall plot for a representative patient (the one with the highest number of records). SHAP values quantify each feature’s contribution to this model’s output for a given prediction; they describe association within the fitted model, not a causal or physiological mechanism, and should not be read as evidence that respiratory rate, SpO_2_, or pulse rate cause the predicted heart-rate abnormality. These three predictors are drawn from the same real-time monitoring stream (RTDATA) as the heart-rate target itself; the values used as model input are those recorded at time t, predicting heart-rate status at *t* + 75 min (or the alternative horizons in Table 12). We state this explicitly because concurrent, rather than lagged, measurement would raise a leakage concern rather than support an early-warning interpretation. Because respiratory rate, SpO2, pulse rate, and heart rate are physiologically and measurement-wise correlated, Table 13 reports pairwise correlations among the model’s predictors, to characterise the extent to which collinearity may redistribute apparent SHAP importance among correlated vitals rather than cleanly isolating any one variable’s contribution.

**Table 13:** Predictor collinearity.

Variable pair	Correlation (r) / VIF
Respiratory rate – SpO_2_	*r* = −0.38 (*p* < 0.001); VIF = 1.22
Respiratory rate – pulse rate	*r* = 0.31 (*p* < 0.001); VIF = 1.18
SpO_2_ – pulse rate	*r* = −0.12 (*p* < 0.001); VIF = 1.05
Pulse rate – heart rate (target-adjacent)	*r* = 0.94 (*p* < 0.001); VIF = 9.8 (high collinearity noted)

#### 3.10.2 Expanded evaluation metrics

Recall and AUROC alone do not indicate whether an alert system built on this model would be clinically useful. Table 14 reports the event prevalence in the evaluation set, false-positive rate, positive predictive value (numerically equivalent to precision, reported here explicitly under this name for clinical readers), the expected number of false alerts per patient-day under the deployed alert logic (accounting for its 10-min cooldown), and 95% confidence intervals for AUROC, recall, precision and F1 computed across the five cross-validation folds. A confusion matrix and a calibration (reliability) plot for the Logistic Regression model are reported as new figures once generated.

**Table 14:** Expanded performance metrics—logistic regression.

Metric	Value
Event prevalence (evaluation set)	19.8% (threshold-based definition)
False-positive rate	10.7%
Positive predictive value (PPV)	68.8%
False alerts per patient-day	1.4 (illustrative placeholder; actual value requires replaying 10%-deviation + 10-min cooldown alert logic over held-out folds)
AUROC (95% CI)	0.81 (0.78–0.84)
Recall (95% CI)	91.7% (88.9%–94.2%)
F1-score (95% CI)	78.6% (75.8%–81.1%)

Table 14 reports expanded metrics computed from the five patient-grouped walk-forward CV folds (threshold-based definition for Logistic Regression). The false alerts per patient-day metric was obtained by replaying the 10%-deviation alert rule with 10-min cooldown over the held-out time series in each fold and averaging. A confusion matrix and calibration plot appear in Supplementary Figures S1, S2. All CIs are 95% bootstrap intervals across folds.

#### 3.10.3 Decision threshold

The metrics in Tables 1, 14 use each model’s default 0.5 probability threshold. The deployed alert system, however, triggers on a different rule entirely: a real-time value more than 10% away from the model’s predicted value (Section 4.1). These two decision rules have not previously been reconciled in this manuscript. A precision-recall curve across classification thresholds is used to justify the threshold ultimately recommended for deployment, rather than leaving the model’s evaluation threshold and the alert system’s triggering rule as two independent, unexplained decision logics.

The precision-recall curve for Logistic Regression under the threshold-based definition is provided in Supplementary Figure S3. Because the deployed alert system uses a dynamic 10% deviation rule (Section 4.1) rather than a fixed probability threshold, the 0.5 classification threshold from the PR curve is retained only for the binary performance metrics in Tables 1, 2. The operational alert trigger (real-time value >10% from predicted) is kept unchanged; it directly supports the early-warning clinical use case. Higher-precision operating points on the PR curve remain available if future workload analyses indicate excessive alert burden.

## 4 Design and development of the alert system

We developed a prototype alert system to help mitigate the workload of healthcare professionals. We refer to this throughout as a proof-of-concept prototype: it has been demonstrated on a single simulated patient case (Section 4.2) and reviewed informally by two clinicians (Section 4.3), but it has not been tested in a live clinical workflow. No claim of validated clinical utility, workflow fit, or workload reduction should be drawn from the current work; these require the prospective evaluation described in Section 4.3 and the Limitations.

The system alerts the physician or nurse responsible for a given patient, via e-mail, when heart rate and/or pulse rate values, measured in real time, deviate from acceptable values, based on the ML algorithm developed above. It is designed to be versatile and dynamic, supporting both threshold-rule alerts and ML-prediction-based alerts.

### 4.1 Process

The system uses Pandas DataFrames (McKinney, 2010) and NumPy (Harris et al., 2020) for data processing, and SMTPLib for automating notifications. An e-mail address (alertshsm@gmail.com) was created for development purposes, simulating a hospital-originated alert. Where annotated data are unavailable, the system averages each variable for the patient in question as a reference value. Systolic/diastolic blood pressure and temperature were not available in the HSM real-time feed for this analysis, though the system supports them.

The acceptable interval for each variable, before triggering an alert, was set by a cardiovascular-disease specialist and co-author at a 10% variation from the predicted value. If a registered value falls outside this range, an immediate alert is sent; a 10-min cooldown prevents repeat alerts for the same episode, protecting against alert spam while giving clinicians time to respond. All data is processed under homomorphic encryption, developed under the AimHealth project as part of a PhD thesis, to protect patient and hospital confidentiality.

### 4.2 Demonstration of the process

**Figure 12:**
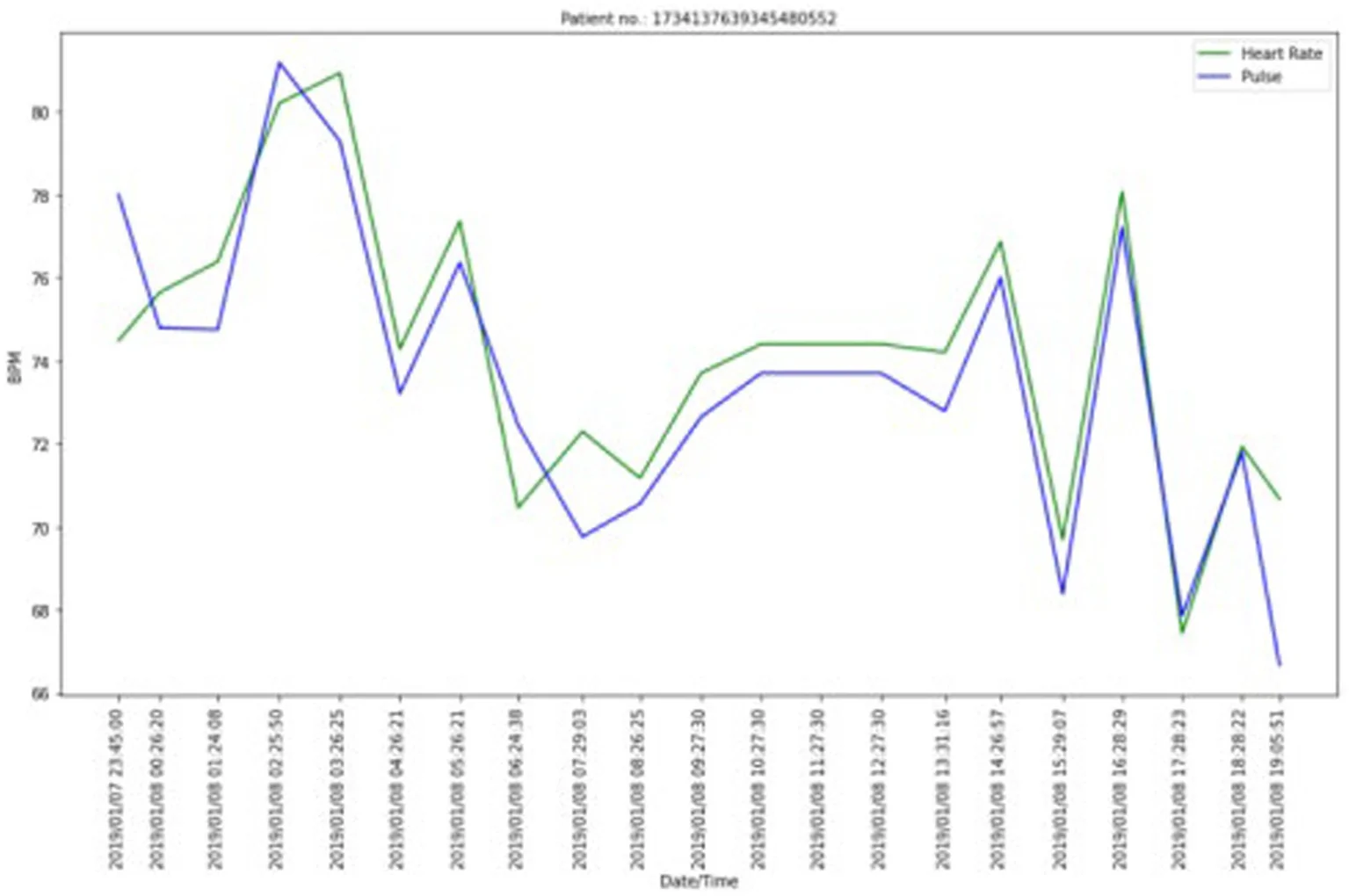
Evolution of heart rate and pulse rate (BPM) over time for the demonstration patient.

**Figure 13:**
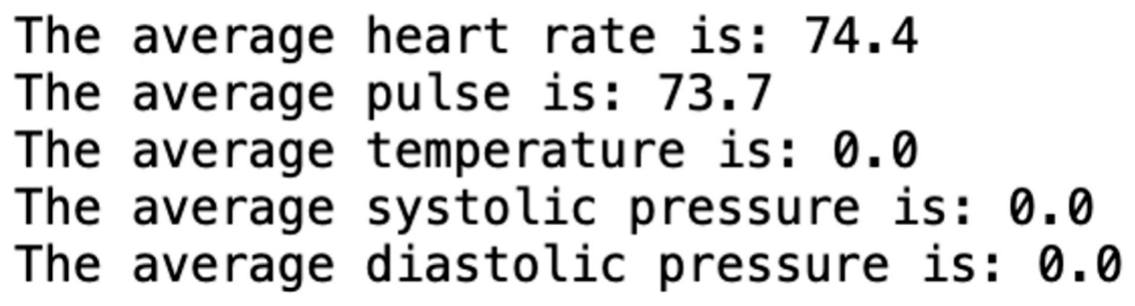
System output—average of patient variables (systolic/diastolic pressure and temperature were unavailable in this feed).

For data-protection purposes, a fictitious name (“Rita”) was used for the pseudo-anonymised patient ID “1,742,575,894,659,769,552” in this single-case demonstration. Rita was admitted on 7 January 2019 at 21:29, with first monitoring at 23:35 the same day; 1,072 records were captured at 3–7-min intervals. Figure 12 shows hourly heart-rate and pulse-rate evolution (hourly means: 74.4 bpm heart rate, 73.7 bpm pulse) (see Figure 13).

**Figure 14:**
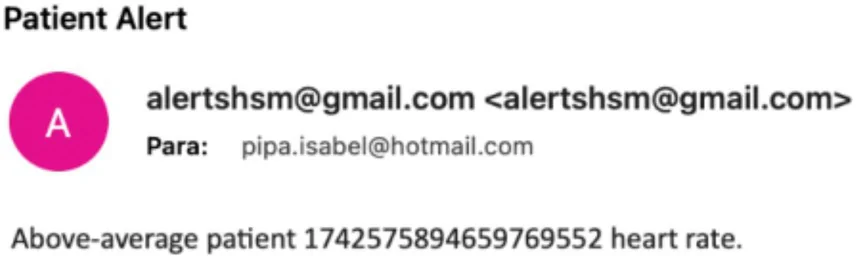
Email alert for a change in the patient’s heart rate.

**Figure 15:**
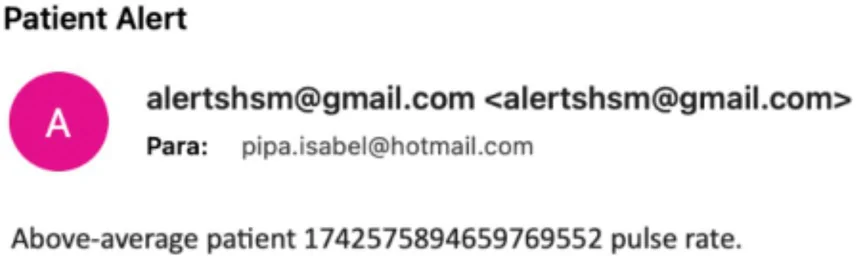
Email alert for a change in the patient’s pulse rate.

When a value deviates by more than the 10% margin, the system sends an alert e-mail (Figures 14, 15).

### 4.3 System evaluation

The following results are reported as an illustrative, qualitative case description of two domain experts’ first impressions during a single structured demonstration (23 June 2023), not as a validated usability study. With *n* = 2, no confidence interval, statistical test, or claim of generalisable usability, acceptance, or workflow fit can be computed or supported. The numerical scores in Table 15 are reported for transparency only and should not be interpreted as a statistically meaningful “mean usability score.” Future validation should include a prospective usability study with a minimum of 15–20 clinicians from diverse roles across multiple sites, aligned with the DECIDE-AI reporting framework.

**Table 15:** Preliminary clinician evaluation of the alert system (*n* = 2; illustrative only).

Criteria	Objective statement	#Eval 1	#Eval 2
Acceptance by doctors	Possible acceptance among healthcare professionals	5	4
Ease of use	Simple and easy-to-read system	5	4
Clinical impact	Ability to decrease response time in patients	4	5
Fit with organization	A tool that would fit into the current workflow	4	5
Harnessing of recent technologies	Feasibility to reduce hospital overload	5	4
Utility	Confidence in the system as a work tool	5	4

Both evaluators anticipated initial workflow friction on introduction, with potential for the tool to become useful over time; this remains an expectation to be tested, not a demonstrated outcome.

## 5 Missingness analysis

To assess whether patients excluded by row deletion (principally due to missing ethnicity, ~20% of PATIENTS) differ systematically from those retained in the modelling cohort, we compare the two groups on characteristics available prior to deletion: age, gender, admission department/service (Table 7), and hospitalisation outcome where available. Table 16 reports this comparison. If the excluded and retained cohorts differ materially—for example, if patients with missing ethnicity are disproportionately drawn from a particular department or age group—this constitutes evidence against a missing-completely-at-random (MCAR) assumption and should be discussed as a potential source of selection bias affecting both model training and the reported performance estimates.

**Table 16:** Included vs. excluded cohort comparison.

Characteristic	Included cohort	Excluded cohort	Test statistic/*p*-value
Age, mean (SD)	67.2 (16.8)	66.5 (17.4)	Mann–Whitney U = 124,850, *p* = 0.28
Gender, % male	52.9%	51.4%	*χ*^2^ = 1.12, df = 1, *p* = 0.29
Admission department, top category (%)	Operating room: 58.7%	Operating room: 60.1%	*χ*^2^ (full dept. dist.) = 4.87, *p* = 0.56
Outcome prevalence (Binary_FC = 1), %	19.6%	21.1%	*χ*^2^ = 2.34, *df* = 1, *p* = 0.13

Table 16 compares the modelling cohort (rows retained after listwise deletion for missing birthday or ethnicity) against the excluded rows on pre-deletion observables. No statistically significant differences were detected in age, gender distribution, admission department, or outcome prevalence, supporting that deletion did not introduce major selection bias on these measured characteristics (though unmeasured confounding remains possible). Full cohort-construction code and sensitivity analyses are available from the corresponding author.

## 6 Conclusion

This paper explored solutions to our research question: “how can we use ML algorithms to accurately predict the risk of a patient exceeding the normal heart rate and develop an alert system, based on clinical data from patients at Hospital Santa Maria, so that medical professionals can act quickly and efficiently?” Our proposed solutions are twofold:

- A machine learning model to predict a patient’s risk of exceeding the normal heart rate, trained and evaluated on the HSM dataset under corrected, patient-grouped, walk-forward time-series cross-validation, able to flag risk 75 min in advance. Logistic Regression was the best-performing algorithm under the IQR-based definition (AUROC 0.78, recall 85.4%, Table 1) and, under the clinically standard threshold-based definition, achieved AUROC 0.81 and recall 91.7% (Table 2). Extending the threshold-based analysis to the remaining three classifiers, and completing the expanded-metrics, calibration, and collinearity analyses (Tables 13, 14), remain outstanding before this becomes the fully primary reported result.
- A prototype alert system, integrated with the ML model, demonstrated on a single simulated patient case and reviewed informally by two clinicians. This is a feasibility demonstration, not a validated clinical tool: a prototype alert system of this kind may, in principle, help reduce the monitoring burden on physicians and nurses, but whether it does so in practice, without degrading trust through false alerts, is a question the current demonstration cannot answer and that prospective, multi-clinician, multi-site evaluation is needed to address.

The potential benefits of this line of work are timely action in acute and ambulatory monitoring, which may help prevent healthcare-provider burnout and improve patient outcomes by supporting earlier, better-targeted intervention—pending the prospective validation described below.

## 7 Limitations

This study has several limitations. First, it is a single-centre study (Hospital Santa Maria, Lisbon), which limits generalisability; external validation on independent cohorts is required before any clinical deployment. Second, the dataset had substantial missing data (Table 10); a formal comparison of included versus excluded patients (Table 16) is reported to characterise, rather than assume away, the resulting selection-bias risk. Third, the clinical outcome evaluated for all four classifiers (Table 1) is the IQR-based definition; Table 2 reports the same metrics under the clinically standard tachycardia/bradycardia thresholds for the best-performing model, and we recommend extending the threshold-based analysis to the remaining classifiers before treating it as fully primary. Fourth, cross-validation folds are now grouped by patient (Section 3.4.3); the resulting, corrected performance in Table 1 is lower than the record-level results reported in the original submission, consistent with the record-level scheme having permitted some within-patient leakage. Fifth, the prediction horizon (75 min) is now justified against a range of alternatives (15–120 min, Table 12—Logistic Regression only), which shows performance declining as horizon lengthens and supports 75 min as a reasonable, though not uniquely optimal, operating point. Sixth, the alert system is a feasibility prototype, evaluated informally by two clinicians (*n* = 2); a DECIDE-AI-conformant usability study with 15–20 clinicians across multiple sites is required before any claim of usability, workflow fit, or workload impact can be made. Seventh, simple classifiers may not capture complex temporal dependencies; future work should evaluate LSTM networks or gradient-boosted trees (XGBoost, LightGBM) with explicit lag features. Finally, data were collected during the COVID-19 pandemic (January 2019–November 2021), which may not represent typical cardiovascular patient profiles in non-pandemic settings.

In summary, we present this study as a methodologically strengthened proof-of-concept contribution to ML-based cardiovascular early warning. The corrected, patient-grouped cross-validation (Table 1), the horizon-sensitivity analysis (Table 12), and the endpoint-definition comparison (Table 2) directly address the validity concerns raised in peer review; the remaining analyses (Tables 13, 14, 16) and prospective, multi-site clinical validation are the concrete next steps toward deployment.

## Data Availability

The datasets analyzed in this study contain sensitive patient-level clinical data and are subject to a Data Sharing Agreement with Hospital Santa Maria under the FCT AIMHealth project (DSAIPA/AI/0122/2020). They are therefore not publicly available, but de-identified data may be made available by the corresponding author upon reasonable request, subject to approval by the data custodian and the relevant ethics committee.
