# Correction: Machine learning-based early detection of abnormal heart rate in critically ill patients: a real-world clinical dataset study with automated alert system integration

**DOI:** 10.3389/frai.2026.1992698

**Published:** 2026-09-23

**Authors:** Joao C. Ferreira, Luis B. Elvas

**Affiliations:** 1 (ISCTE-IUL) – ISTAR-IUL, Information Sciences, Technologies and Architecture Research Center, University Institute of Lisbon (ISCTE), Lisbon, Portugal; 2 Faculty of Logistics, Molde University College – Specialized University in Logistics, Hogskolen i Molde, Molde, Norway; 3 Department of Logistics, Molde University College, Molde, Norway; 4 INOV INESC Inovação – Instituto de Novas Tecnologias, Lisbon, Portugal; 5 Breast Cancer Research Program, Champalimaud Foundation, Lisbon, Portugal

**Keywords:** cardiovascular monitoring, clinical decision support, critical care, digital health, early warning system, heart rate prediction, machine learning, SHAP explainability

Affiliation Department of Logistics, Molde University College, Britvegen 2, Molde, Norway, INOV INESC Inovação – Instituto de Novas Tecnologias, Lisbon, Portugal and Breast Cancer Research Program, Champalimaud Foundation, Lisbon, Portugal were omitted for author Luis B. Elvas. These affiliations have now been added for author Luis B. Elvas.

The original version of this article has been updated.

